# Lodging resistance of rice plants studied from the perspective of culm mechanical properties, carbon framework, free volume, and chemical composition

**DOI:** 10.1038/s41598-022-24714-4

**Published:** 2022-11-21

**Authors:** Qing Liu, Chongshan Yin, Xi Li, Chunqing He, Zhi Ding, Xuan Du

**Affiliations:** 1 Chinese Academy of Sciences, Institute of Subtropical Agriculture, Changsha, China; 2 Hunan Provincial Key Laboratory of Flexible Electronic Materials Genome Engineering, School of Physics and Electronic Science, Changsha University of Science and Technology, Changsha, 410114 China; 3 Key Laboratory of Nuclear Solid State Physics Hubei Province, School of Physics and Technology, Wuhan University, Wuhan, 430072 China

**Keywords:** Nuclear physics, Biophysics, Materials science, Physics

## Abstract

In this study, four varieties of rice were cultivated on the same farmland under same conditions and for same duration. However, their lodging resistance was found to be obviously different from each other. Herein, three key factors that highly influenced the lodging resistance were identified. First, in terms of morphological trait, in contrast to the generally believed theory that the overall thickness of the fresh culm wall governs the strength, the thickness of the depressed region of the dried basal culm wall largely determined the mechanical properties by acting as the weak link. This depressed region represents the vulnerable part with high syneresis rate. Second, the culm and its carbon framework exhibited sufficient strength and rigidity for both support and stability of the rice stem. The constraint of high lodging resistance of rice plants is attributed to the culm flexibility. Furthermore, the results of the positron annihilation lifetime spectroscopy corroborate that the most amorphous part and the highest-fraction free volume in the culm carbon framework were found for samples that exhibited high lodging resistance. This result confirmed the significant influence of the culm flexibility on lodging resistance. Third, a higher level of nitrogen element content in the basal culm can benefit its growth and development, which may contribute to an increase in lodging resistance of rice plants.

## Introduction

Rice is a staple food for more than half of the world’s population. One of the most concerning problems faced by farmers is the lodging of rice plants near the crop harvest stage, which leads to the decrease in both crop production (about 5–80%) and crop quality, as well as reduction in the mechanical harvesting efficiency^1–3^. Lodging indicates the permanent displacement of vertical plants, which is a result of stem breaking, stem bending, or root lodging^4–6^. Stem lodging occurs when the plant is damaged or the entire plant becomes Euler unstable; however, the root lodging is always caused by the anchorage failure^7^. Many factors determine the lodging resistance of stem, such as plant mechanical performance, unstable external environment, silicon content, soil density, diseases, illumination intensity, external stimuli, natural disasters, and overplant population^8–11^. Owing to the complexity of the lodging phenomenon, there are still many questions that remain to be answered, and how to improve the lodging resistance is a research hotspot in the field of rice breeding over the years^12,13^.

The basal stem of the rice plants consists of culm and leaf sheath. The culm, which is composed of vascular bundles and mechanical tissue layer, plays a dominant role in the lodging resistance of the overall plant. Rice stem contributes around 65.8–95% of the overall bending stiffness of stem segment^14^, and the remaining is attributed to the leaf sheath^15^. The relationship between morphological characteristics and mechanical properties of the basal culm has been extensively studied till date^16–19^. In terms of the mechanical properties of plant, the primary influencing factors are stem diameter, culm wall thickness, leaf sheath, plumpness, dry density, vascular bundles, internode length, plant height, height of the center of gravity, rigidity of the lower portion, weight of the upper portion, etc^4,16–18^. Normally, greater culm diameter and wall thickness are largely favorable for the lodging resistance. Moreover, the carbohydrate components (such as cellulose, soluble sugar, starch, and lignin) of basal culm also contribute to its rigidity^20,21^. The relationship between these traits and rice genotypes (either prone to lodging or displaying lodging resistance) has already been confirmed^22–28^.

Further, fertilization (in particular nitrogen fertilization) is used as one of the major measures for improving grain yield in rice. Previously, extensive research efforts have been devoted to investigate the effects of nitrogen content on rice. Results indicate that excessive nitrogen increased the rice tiller numbers, plant height, and weight of the upper portion, and then it finally led to lodging and yield loss^1,16,17^. Thus, optimized nitrogen management is important for rice cultivation. However, most previous studies mainly focused on the relevance between the total amount of nitrogen fertilizer and morphological and physiological characteristics of rice plants^29,30^. Considering the absorption and utilization of nitrogen fertilizer within rice plants, the distribution of nitrogen in culm significantly impacts the growth and development of the rice plants, which can be very meaningful to better comprehend the lodging resistance.

Rice stem can be regarded as an outer shell consisting of almost fully dense materials supported by a low-density hollow foam core. The outer shell consists of a mechanical tissue layer composed of thick skin fiber cells, and the foam core is made up of a parenchymal tissue reinforced with the vascular bundles^31,32^. It has been extensively studied that the biomechanical factors of outer shell and vascular bundles dominate the stiffness of the stem. These factors include diameter and wall thickness of outer shell, number of vascular bundles, and microfibril angle and thickness of the sublayer of cell wall^29–34^. However, comprehensive understanding of the effect of lodging resistance at the level of micro-scale or ultra-scale structures is still unclear^33,34^. In particular, it is considered that the physical properties of culm influenced by its carbon framework are significantly important in correlating with its mechanical properties. Carbon atoms create the backbone of living matter, which is denoted as the carbon framework. Free volumes are abundant in the carbon framework, which refer to the microscopic area of a substance that is not occupied by atoms and electrons^35–38^. The size, distribution, and density of free volumes are closely related to the macroscopic properties of matters, such as mechanical properties, glass transition, thermal expansion and contraction, and relaxation phenomenon^39–42^. Thus, based on perspective of free volumes, intensive studies regarding the mechanism behind the mechanical properties of the rice culm can be interesting and of utmost importance. On the other hand, it is extremely difficult to observe the free volumes in the solid matters by using conventional techniques, such as transmission electron microscopy or atomic force microscopy. For several decades, positron annihilation technique has been widely used for the characterization of open volumes, vacancy type defects, and pores in various materials^39–43^. Thus, in this study, positron annihilation lifetime spectroscopy (PALS) was employed to characterize the free volume holes within the carbon framework of the rice culm in order to better comprehend the effect of free volumes on the mechanical strength and lodging resistance of rice plants. Herein, four varieties of rice were cultivated on the same farmland under same conditions and for same duration; however, their lodging resistance was obviously different. The reasons behind this phenomenon were investigated in detail in terms of the culm mechanical properties, morphology, carbon framework, free volumes, and chemical composition.

**Figure 1:**
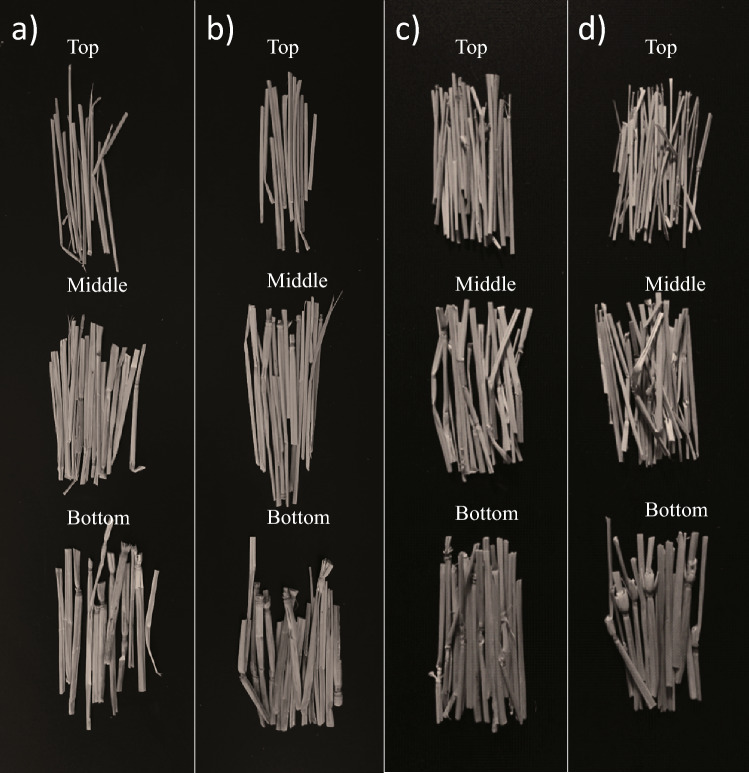
Pictures of the culm of (**a**) R1, (**b**) R2, (**c**) R3, and (**d**) R4. The culm was cut into 3 parts: top, middle, and bottom (or basal).

## Materials and methods

### Materials

Field experiments were conducted in Changsha, Hunan province, China (113.16 E, 28.16 N) during the rice-growing season. Four different varieties of cultivated rice plants (Xianghancan45, Jusui, Lixiangzhan, and Aipei) were grown on the same farmland with the same planting density, seeded, and harvested from the end of March to mid-August, or mid-April to end-August, 2021. All rice plants were loosely planted (15-16 plants per square meter) to reduce the effect of additive disturbances. There are no significant climate differences during the rice growth period, and the duration of the growth period is basically the same (4.5 months). For each variety of rice, 10-20 main stems from rice plants were randomly collected from the farmland. Before the measurement, dirt on all plants has been carefully washed off.

### Preparation of the rice culm and carbonized rice culm

First, the root, leaf, and spike of all rice plants were cut off (as shown in Fig. S1). Then the fresh spike was weighted. Second, the culm was taken out from the basal stems (consist of culm and leaf sheath) and weighted. The culm was cut into three parts, namely, top, middle, and bottom (or basal). The top part refers to the culm beyond the topmost node and the bottom part refers to the culm between the lowermost node and the root, and the middle part means all the rest of the top and bottom part. The photograph of the 3 parts is shown in Fig. 1. Third, the resulting culms were dried at 45 $^{\circ }$C for 72 h, and weighted again. After the drying treatment, the culm wall with uniform thickness becomes uneven. Fourth, some culms were cut into small pieces and then processed into powder with a grinder. Subsequently, all of the resulting culm powder was screened with a filter sieve. Finally, some of the culm powder was placed on a porcelain boat and transferred to a horizontal tubular furnace. Under N$_2$ atmosphere, the samples were heated to 400 $^{\circ }$C and kept warm for 1 h for the carbonization process. The culm powder and carbonized culm powder were then stored in a drying cabinet.

### Characterization

For measuring the lodging degree, rice plants were grown separately by variety, and arranged into elongated rectangles (about 200 rows and 10 columns). The lodging degree refers to the number ratio of plants lodging at angle greater than 45$^{\circ }$ against the overall plants. For each rice variety, the resulting lodging degree was based on the analysis of a total of 100 samples randomly collected from the field, at the maturing stage and before the harvest. Very small number of artificially damaged and diseased rice plants was not included.

The morphologies of the carbonized culm were measured by a Scanning Electron Microscope (SEM) (Sigma HD, Carl Zeiss Jena, Germany). The mechanical properties (maximum bending moment) of the basal culm were measured on a tensile machine (HDV, Yueqing Handpi Instruments, Co., Ltd., China). The crystallinity of the samples was characterized by X-ray diffraction (XRD) and X-ray powder diffraction (XRPD) measurements, performed on a D8 ADVANCE type diffractometer (Bruker, Germany), with the scan rate of 4$^{\circ }$ min$^{-1}$. The chemical elements of the rice culm samples were measured by an elemental analyzer (Vario EL CUBE, ELEMENTAR, Germany). The porosity (surface area) of the samples was characterized by BET method (Brunauer-Emmett-Teller) via N$_2$ adsorption-desorption process (JW-BK 122W, JWGB, China) at 77 K. The surface morphologies of the fresh rice culm were measured by a metallographic microscope (NJL-120A, Beijing Novel Optics Co., Ltd., China ). The X-ray photoelectron spectroscopy (XPS) measurement was conducted for all rice culms in powder form (K-Alpha, Thermo Scientific, USA ). The Fourier Transform Infrared Spectroscopy (FTIR) was carried out at room temperature at a resolution of 2 cm$^{-1}$ by a Nicolet 170 SXIR spectrometer (Thermo Scientific, USA) in the range of 4000 cm$^{-1}$ to 500 cm$^{-1}$. Free volume holes within the carbonized rice culm were studied by a positron annihilation lifetime spectroscopy (PALS) technology and a mechanical property measurement. The measurement was conducted by using a fast-fast coincidence PALS with a time resolution function of 0.230 ns for the full width at half maximum (FWHM), and 1 million counts were collected for each spectrum. Details about the PALS measurement can be found in the Supporting Information.

In this study, the statistical data of sample is presented as an average value (AV) and a standard deviation (SD) as “AV ± SD”. Then, based on the Welch’s t-test, the P-value along with related data is used to represent the significant difference in data when needed. For each rice variety, the lodging degree was obtained based on 100 plants, the maximum bending moment measurement was conducted three times, the traits value of plants presented in Table 2 was measured at least 10 times, and the traits value of plants presented in Table 3 was measured at least 50 times. The results are presented as the average values.

**Table 1:** Maximum bending moment M$_{max}$ of fresh culms; Lodging resistance and lodging degree of rice plants.

Label	Variety	$M_{max}$ (N mm)	Lodging resistance	Lodging degree (%)
R1	Xianghancan	53± 10	Best	0
R2	Lixiangzhan	49± 8	Good	9
R3	Jusui	50± 13	Normal	55
R4	Aipei	48± 8	Worst	80

The symbol ± in table refers to the distribution range of data.

### Author statement for the use of plants

In this study, collection and use of plant material comply with relevant institutional, national, and international guidelines and legislation.

## Results and discussion

### Mechanical property and morphology of fresh culm

The four different rice cultivars, namely, Xianghancan 45, Lixiangzhan, Jusui, and Aipei (labeled as R1, R2, R3, and R4, respectively), were grown on the same farmland under the same growing conditions. However, their lodging resistance was obviously different from each other. Both R1 and R2 rice plants exhibited good lodging resistance. More importantly, most of the R1 plants basically remained in a perpendicular position (> 80%). However, R3 and R4 varieties showed a relatively high degree of lodging. In particular, many of the R4 plants exhibited the lodging larger than 80$^{\circ }$ from the perpendicular position (> 30%). In this study, lodging of the rice plants occurred dominantly near the bottom, which was mainly caused by the breaking or bending of the basal stem.

The lodging degree was found to be 0, 9, 55, and 80%, for R1, R2, R3, and R4, respectively, as presented in Table 1. Therefore, the lodging resistances of the four varieties of rice plants are in the order of R1> R2> R3 > R4. Basically, the maximum bending moment (M$_{max}$) reflects the rigidity of the culm^44^. Table 1 summarizes that the M$_{max}$ of R1 is slightly higher than that of the others, and no linear relationship was observed between the lodging resistance and the M$_{max}$. This is attributed to the fact that the lodging of rice plants is an extremely complex process and it is influenced by several factors^31,32^. Furthermore, by considering the significant difference in lodging resistance of the R1, R2, R3, and R4 rice plants, surprisingly, it was found that the M$_{max}$ values of the samples were fairly similar to each other. Thus, the rigidity of the culm is only one of the factors among many that is associated with plants lodging, and probably not the major one. Therefore, undeniably, more experimental studies are still required to obtain a more comprehensive understanding of this problem of lodging.

**Figure 2:**
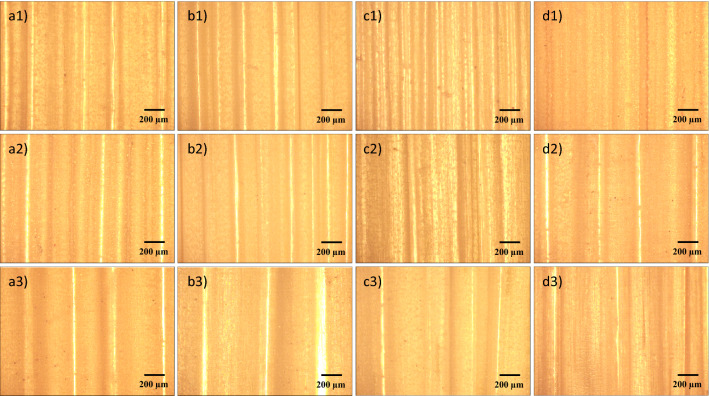
The surface morphologies of culm wall of (**a**) R1, (**b**) R2, (**c**) R3, and (**d**) R4. The numbers 1, 2, and 3 in figure indicate that the morphologies were taken from the top, middle and bottom of the stem wall, respectively.

**Figure 3:**
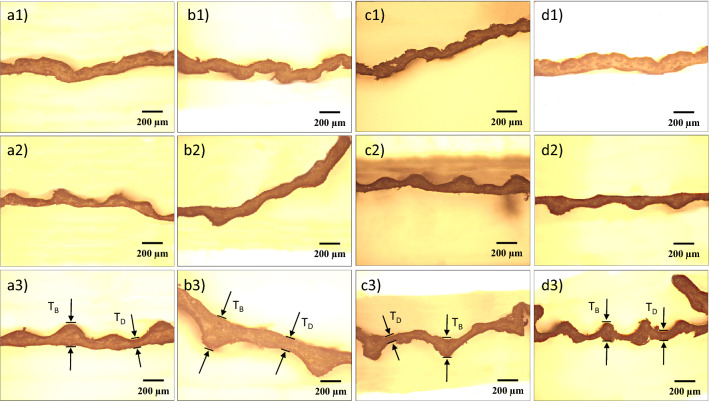
The cross section morphologies of culm wall for (**a**) R1, (**b**) R2, (**c**) R3, and (**d**) R4. The numbers 1, 2, and 3 indicate that the morphologies were taken from the top, middle and bottom of the culm wall, respectively. Examples for the bulge region and the depressed region have been marked with arrows and lines.

**Table 2:** The number of samples (N), average length (L$_{Culm}$), weight (W$_{Culm}$), length density (LD$_{Culm}$) for a single culm.

Label	N	$L_{Culm}$ (cm)	$W_{Culm}$ (g) fresh/dry	$LD_{Culm}$ ($10^{-1}$g/cm) fresh/dry	T (µ m) fresh	$W_{Spike}$ (g) fresh	$H_{CG}$ (cm)	Culm node
R1	20	65±6	10.94/2.28	1.68/0.35	232±35	5.16	42.9	3-4
R2	10	66±4	11.13/2.25	1.69/0.34	251±39	5.03	43.3	3-4
R3	10	68±5	11.76/2.43	1.73/0.36	237±30	4.80	43.9	3-4
R4	20	59±8	9.92/2.08	1.68/0.35	234±27	3.63	37.4	3-4

The wall thickness (T) of the fresh basal culm. The fresh spike weight (W$_{Spike}$), height of center of gravity (H$_{CG}$), culm node of rice plants. The symbol ± in table refers to the distribution range of data.

**Table 3:** Diameter of the top (D$_{Top}$), middle (D$_{Middle}$) and bottom (D$_{Bottom}$) of the fresh culm.

Label		R1	R2	R3	R4
$D_{Top}$ (mm)	Range/average	2.2-2.8 / 2.4	2.1-2.6 / 2.3	2.3-2.6 / 2.5	2.1-2.7 / 2.4
$D_{Middle}$ (mm)	Range/average	4.2-4.7 / 4.5	4.2-4.7 / 4.4	4.4-4.7 / 4.6	4.3-4.6 / 4.4
$D_{Bottom}$ (mm)	Range/average	4.9-6.0 / 5.7	5.1-5.9 / 5.6	5.2-6.2 / 5.8	4.7-6.0 / 5.6
$T_{B}$ (µ m)	Top	154±7	151±10	143±11	174±13
	Middle	157±19	166±23	165±21	173±20
	Bottom	209±34	222±60	211±29	203±43
$T_{D}$ (µ m)	Top	105±14	86±6	84±14	133±12
	Middle	84±12	97±5	86±9	79±4
	Bottom	93±5	118±12	70±4	61±12
$T_{B}$ / $T_{D}$	Top	1.47	1.76	1.70	1.31
	Middle	1.87	1.71	1.92	2.19
	Bottom	2.25	1.88	3.01	3.33

The thickness of the bulge region (T$_{B}$) and thickness of the depressed region (T$_{D}$) of the dried culm wall measured by a metallographic microscope. The symbol ± in table refers to the distribution range of data.

Plants lodging normally involves morphological features, anatomical traits, and chemical composition of the fresh culm^9–13^. To find reasons for the difference in the lodging resistance of the samples, related traits of the plants were obtained and summarized in Table 2. The wall thickness values of the fresh basal culm (T) of samples are close to each other (232–251 $\mu$m), and show no obvious regularity as a function of lodging resistance. Culm lengths for R1, R2, and R3 are basically similar (65–68 cm), while that for R4 is shorter (59 cm). The length density of all culms is almost the same (1.68–1.73 and 0.34–0.36 10$^{-1}$ g cm$^{-1}$ for fresh and dry culm, respectively), and the number of culm nodes is 3-4 for all samples. The spike of R1 (5.16 g) and R2 (5.03 g) is heavier than that of R3 (4.80 g) and R4 (3.63 g). Moreover, no significant difference was noticed in the H$_{CG}$ for R1, R2, and R3 (42.9 - 43.9 cm), and that of R4 was even lower (34.7 cm). Consequently, in this study, plant height, spike weight, fresh culm wall thickness, culm length density, and height of the center of gravity of plant are not the primary factors that determine the plant lodging resistance. In this case, it is predicted that the mechanical properties of the basal culm likely play a dominant role in the lodging resistance of the samples^2,31^.

In order to reveal how the mechanical properties of the culm determine the lodging resistance, the diameter of fresh culm, which has been widely recognized to correlate with the lodging resistance of crops^33,45^, was obtained and the corresponding results are summarized in Table 3. However, in this study, no significant difference in D$_{Bottom}$ was found among samples (ranging from 5.6 to 5.8 mm). To further analyze the structure of the culm, the metallographic surface and cross-sectional morphologies of the dried culm wall were obtained, as shown in Figs. 2 and 3, respectively. Apparently, wrinkles along the culm could be found in all samples (Fig. 2), which resulted from the uneven thickness of dry culm walls (Fig. 3). Basically, the closer the wrinkles are to the bottom, the larger they are in size and greater in separation distance, because of the increase in culm radius. This uneven thickness of dry culm walls is caused by the different syneresis rates of vascular bundles and mechanical tissue layer within the culm^31^. The depressed region represents the part of culm structure that is vulnerable and with high syneresis rate. Furthermore, the wall thickness of dried culm was measured, and the results are presented in Table 3 . The thickness of the bulge region (T$_{B}$) for basal culm (203–222 $\mu$m) shows no obvious regularity as a function of lodging resistance of samples. The thickness of the depressed region (T$_{D}$) for basal culm of R1 and R2 (93 and 118 $\mu$m, respectively) is evidently higher than that of R3 and R4 (70 and 61 $\mu$m, respectively). This significant difference was confirmed by the Welch’s t-test (P-value < 0.01). Furthermore, the specific value of T$_{B}$/T$_{D}$ ratio for basal culm of R1 and R2 (2.25 and 1.88, respectively) is also higher than that of R3 and R4 (3.01 and 3.33, respectively). Thus, in contrast to the generally believed theory that the overall thickness of the fresh culm wall governs the strength^34^, it is likely that the depressed region of the dried basal culm wall largely determined the mechanical properties of the culm. This depressed region probably acts as the handicapped area for the mechanical properties of the overall culm.

**Figure 4:**
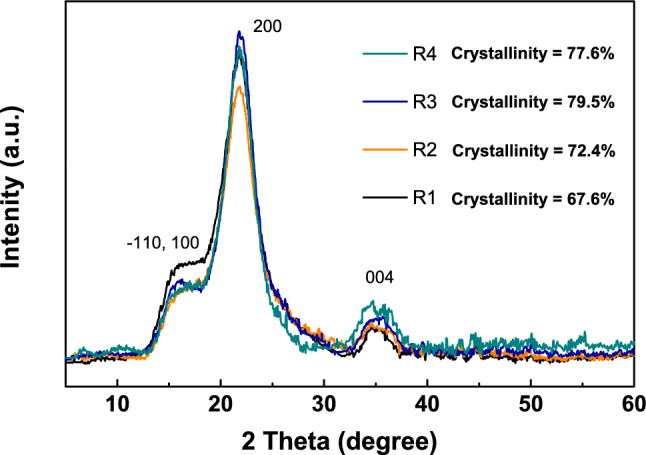
XRD patterns of the basal rice culms, which have been reduced into a fine powder before measurement.

### Components and crystallinity of fresh culm

In terms of composition, the mechanical properties of the culm primarily depend on its carbohydrate components, such as cellulose, starch, soluble sugars, and lignin^17^. For rice, the cellulose content mainly determines the physical strength of the stem by serving as a link and support^20,21^. To confirm this phenomenon, physical and chemical characterizations were conducted, by using XRD, and FTIR, respectively. Figures 4 and S2 show the XRD patterns and FTIR spectra of the powdered basal culm of the samples, respectively. Both the XRD and FTIR results suggest a typical cellulose I structure^46,47^. In Fig. 4, three major diffraction peaks have been noticed for all samples, and they were assigned as the crystalline planes in the crystal structure of cellulose I. Thus, there is no difference in the type of cellulose among the samples. In detail, the peak at 2$\theta$ = 16.5$^{\circ }$ represents a composite peak of the crystalline planes with Miller indices of -110 and 110. On the other hand, peaks at 2$\theta$ = 21.8$^{\circ }$ and 35.0$^{\circ }$ indicate the crystalline planes with Miller indices of 200 and 004, respectively. According to these peaks, the crystallinity of the cellulose has been obtained and it is different among the samples.

Normally, higher crystallinity of cellulose indicates that the nano-cellulose fibrils approach each other closely and form tight aggregates, which results in a more rigid structure and better mechanical strength^46^. Therefore, it is generally believed that higher crystallinity of cellulose is beneficial to the lodging resistance of rice. However, in this study, although not demonstrating a simple linear relation (as shown in Fig. 4), it is found that the lower crystallinity of cellulose basically guarantees a better lodging resistance of the rice plants. A similar result has been reported by Li et al. in 2015^48^. In that work, Li et al. performed systems biology analyses of a total of 36 distinct cell wall mutants of rice, and results suggest that the cellulose crystallinity was the key factor that negatively determines the lodging resistance in rice plants. The hemicellulosic arabinose was detected to be the major factor that negatively affects cellulose crystallinity probably through its interlinking with $\beta$-1, 4-glucans. In this case, a lower cellulose crystallinity benefits the mechanical properties of rice culm against lodging. It is likely that, rather than the rigidity properties, the flexibility of the culm is more important for determining the lodging resistance of plants, because higher crystallinity leads to a lower elongation as well as poorer flexibility of the plant stem^49^. This conclusion is consistent with the above-mentioned results that show the M$_{max}$ values of the samples are fairly close to each other (see Table 1), while their lodging resistance is obviously different from each other. The influences of stem flexibility on plants lodging resistance were further studied from the perspective of the porous structure and free volumes of the culm carbon framework, which are discussed in the subsequent sections.

The crystallinity indices of the powdered rice culm were obtained directly from the height ratio between the intensity of the crystalline peak (I$_{200}$–I$_{Amorphous}$) and total intensity (I$_{200}$)^49^. In particular, it should be noted that the resulting crystallinity of the cellulose is a relative one, because the three diffraction peaks in Fig. 2 are composite peaks^46^. For each peak, the assigned crystalline plane contributes the major portion, and several other crystalline planes overlap slightly. According to the reported literatures, the real value of the cellulose crystallinity is not significantly affected by this situation^46^. Thus, the resulting crystallinity is reliable for comparison purposes.

**Table 4:** Elemental analysis [carbon (C), oxygen (O), nitrogen (N), and hydrogen (H)] of the culms.

Label	C (wt.%)	O (wt.%)	N (wt.%)	H (wt.%)
	Bottom	Bottom	Top/Middle/Bottom	Bottom
R1	37.80	40.69	1.37/1.70/1.66	6.02
R2	37.37	41.00	1.31/1.62/1.57	5.93
R3	37.88	40.30	1.79/1.25/1.20	5.80
R4	37.16	38.54	1.89/1.20/1.24	5.78

**Figure 5:**
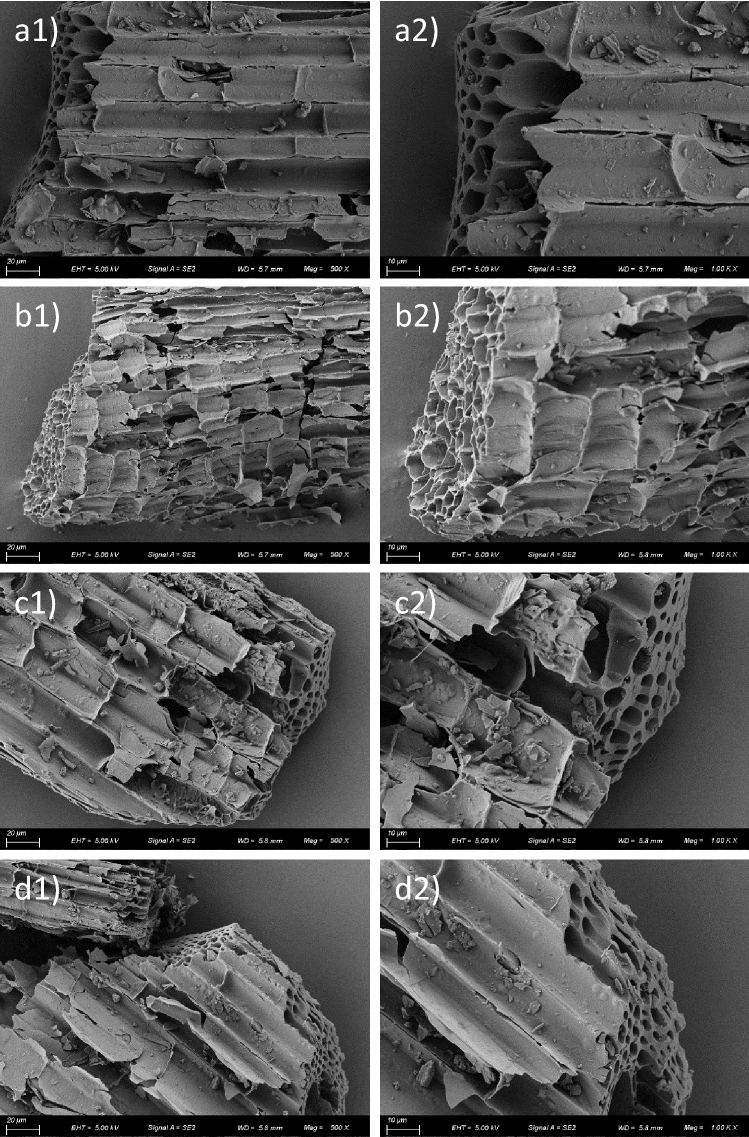
SEM images of the carbonized basal culms of (**a**) R1, (**b**) R2, (**c**) R3, and (**d**) R4. The (**a2**), (**b2**), (**c2**), and (**d2**) are the 2 times magnified images of (**a1**), (**b1**), (**c1**), and (**d1**), respectively. The scale has been shown at the lower part of each image.

**Figure 6:**
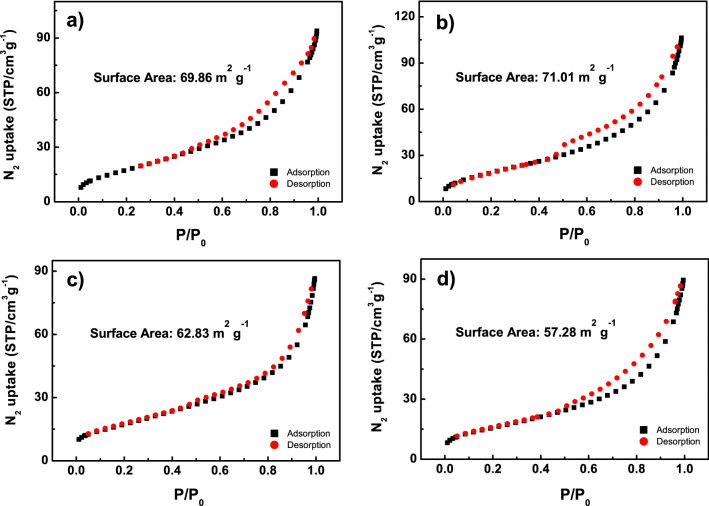
N$_2$ adsorption—desorption isotherms of the carbonized basal culm (in powdery) of (**a**) R1, (**b**) R2, (**c**) R3, and (**d**) R4.

### Element analyze of fresh culm

The result of elemental analysis of the culm is presented in Table 4. There is no evident difference in the content of C, O, and H among the samples. On one hand, for R1 and R2, the middle and bottom regions of the culm show a higher level of N content, while on the other hand, the higher level of N content for R3 and R4 is found in the top region. For the bottom region, N content for R1 and R2 is 27 - 38% higher than that for R3 and R4. For the middle region, N content for R1 and R2 is 30–42% higher than that for R3 and R4. Normally, N content plays an important role in rice production^29,30^. Both insufficient and excessive nitrogen application can have an unfavorable impact on rice production, leading to the reduction of rice yield and quality, and may bring lodging risk^1^. For example, it is well known that nitrogen performs obvious function in the photosynthesis and metabolism of plant. Nonetheless, excessive nitrogen may decrease the culm diameter and wall thickness, plumpness and bending strength, and degree of lignification and mechanical tissue thickness of rice plants^1,16,17^. In this study, it was found that a relatively higher nitrogen element content benefits the growth and development of rice culm. Notably, the lodging of the rice plants occurs primarily near the bottom; therefore, a higher level of N element content in the basal culm may contribute to an increase in lodging resistance of rice plants.

### Porous structure of the carbon framework of rice culm

The morphologies of the carbonized basal culm are shown in Fig. 5. The carbon framework of all samples exhibits a porous structure, and the diameter of those macro-pores is mainly ranging from 1-10 $\mu$m. Because of the complexity and non-uniformity of the microstructure of the culm obtained from natural rice plants, it is not adequate to analyze the porosity of the culm carbon structure only for a small area (as in a $\sim$100 $\times$ 100 $\mu$m$^2$ area, as shown in Fig. 5). Thus, the statistical surface area of the carbonized rice culms (of 0.2 g each) was measured by BET method, and the N$_2$ adsorption-desorption isotherms at 77 K are displayed in Fig. 6. As calculated from the isotherms, the surface area of R1, R2, R3, and R4 is 69.86, 71.01, 62.83, and 57.28 m$^2$ g$^{-1}$, respectively. The lodging resistance of the samples is in the order of R1> R2> R3 > R4, and the BET results indicate that the larger surface area of the carbon framework of the culm is in favor of the lodging resistance. A larger surface area indicates a less tight structure (a more porous structure) of the carbon framework, which contributes to the flexibility of the rice culm^50^. Therefore, the results strongly suggest that, even with a relatively loose arrangement, the carbon framework has sufficient strength and rigidity for both support and stability of the rice culm. It is also important to note that the flexibility of the carbon framework plays an important role in the lodging resistance of the rice culm.

### Free volumes and flexibility of the carbon framework of rice culm

**Figure 7:**
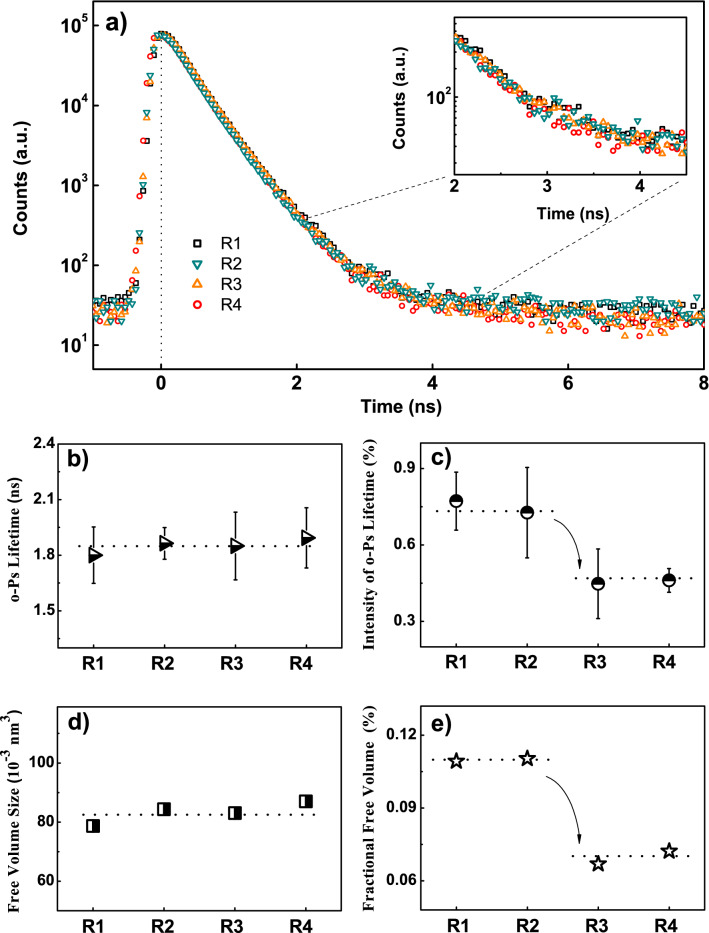
(**a**) Raw positron lifetime spectra of samples; The variations in (**b**) o-Ps lifetime ($\tau$ $_{o-Ps}$), (**c**) the intensity (I$_{o-Ps}$), (**d**) the sizes of free volumes (V$_{FV}$) obtained by using the Tao-Eldrup model, and (**e**) the fractional free volume (FFV) of the rice basal culm. Arrows and dotted lines are guides to the eyes.

PALS characterization has been employed in this research to analyze the microstructure within the carbon framework. The raw positron lifetime spectra of the samples obtained from PALS are displayed in Fig. 7a as the characterization of free volume holes inside the samples. In this study, we focus on the ortho-positronium, which is of particular importance for the studies on free volumes because it is related to the average free volume hole size. Details about the principle of the PALS measurement can be found in the Supporting Information. As shown in Fig. 7, the $\tau$ $_{o-Ps}$ (lifetime of ortho-positronium) and the free volume size (V$_{FV}$) of the samples are almost similar, while the values of I$_{o-Ps}$ (relative intensity of ortho-positronium) and the fractional free volume (FFV) of R1 and R2 (I$_{o-Ps}$ = $\sim$0.75% and FFV = $\sim$0.11%) are higher than those of R3 and R4 (I$_{o-Ps}$ = $\sim$0.45% and FFV = 0.07%). It is important to note that the o-Ps is almost impossible to be formed in defect-free carbon-based materials, such as graphite crystal and graphene crystal^51^. The resulted $\tau$ $_{o-Ps}$, I$_{o-Ps}$ and V$_{FV}$ mainly characterized the free volumes within the amorphous part of the carbon framework, which contains numerous defects. Thus, the similar $\tau$ $_{o-Ps}$ and V$_{FV}$ results indicate the similarity in both microstructure and mechanical property of the amorphous part of the carbon framework among the samples. The decrement in I$_{o-Ps}$ from $\sim$0.75% (R1 and R2) to $\sim$0.45% (R3 and R4) suggests a reduction of the area of the amorphous part (or a decline in the number of the defects) within the carbon framework. Accordingly, a certain amount of amorphous phase is beneficial to the mechanical properties of the carbon framework. In detail, the amorphous phase contributes to the mechanical properties of the carbon framework by improving its flexibility, because the FFV of R1 and R2 ($\sim$0.11%) is significantly higher than that of R3 and R4 ($\sim$0.07%), as shown in Fig. 7e. Higher FFV underlines the higher mobility of the carbon molecular chain in the carbon framework^52,53^. On the macro level, it provides better flexibility to the carbon framework. As a result, from several aspects, data in this study strongly suggest that the culm and its carbon framework exhibit sufficient strength and rigidity for both support and stability of the rice stem. The constraint of high lodging resistance of rice plants comes from the flexibility.

In addition, it is noticed that the value of FFV is low for all samples, which is caused by the fact that the o-Ps can hardly be formed in defect-free carbon material^51^, as previously mentioned. Thus, the actual value of FFV in the samples can be higher to a certain extent. Moreover, the resulted FFV value is an apparent one and can only be used for the comparison among the samples studied in this research. The FFV was calculated according to $\tau$ $_{o-Ps}$ and I$_{o-Ps}$ by following the steps below. Firstly, obtain the average radius of free-volume holes (R) by using the Tao-Eldrup model as^36,37^,

1 $$\begin{aligned} \tau _{o-Ps}=0.5\ \left[ 1-\frac{R}{R{_0}}+\frac{1}{2\pi }sin\left( \frac{2{\pi }R}{R_0}\right) \right] ^{-1}(ns) \end{aligned}$$

where *R*$_0$=*R*+$\Delta$*R*, and $\Delta$*R*=0.166 nm is the thickness of the o-Ps wave function overlapping with the homogeneous electron layer. Then, the V$_{FV}$ can be obtained by,

2 $$\begin{aligned} V_{FV}=\frac{4}{3} \pi R^3. \end{aligned}$$

Finally, the FFV turns out to be the product of the V$_{FV}$, the I$_3$, and a constant C,

3 $$\begin{aligned} FFV=C\ V_{FV}\ I_3, \end{aligned}$$

where the value of C is 0.0018 $\overset{\circ }{A}$ $^{-3}$^54,55^.

### Chemical property of the carbon framework of rice culm

**Figure 8:**
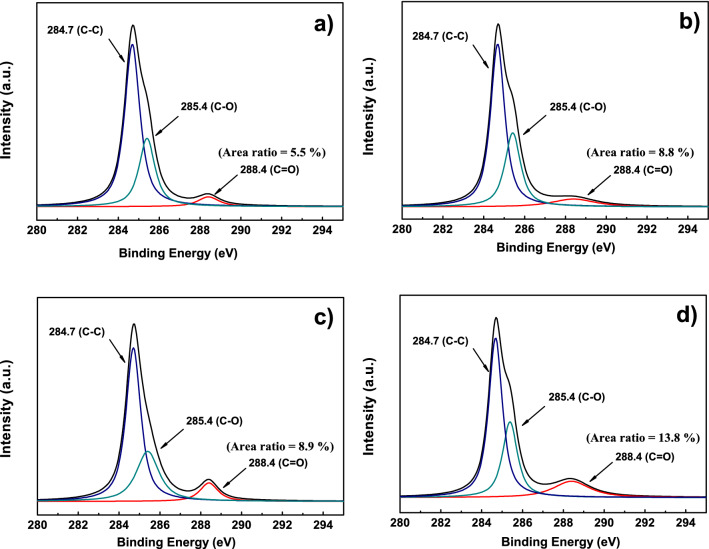
XPS spectra of the carbon framework of rice culm (**a**) R1, (**b**) R2, (**c**) R3, and (**d**) R4. The area ratios of the peak for C=O bonds have been calculated and shown in the figure.

Commonly, the basic mechanical properties of the carbon framework are greatly decided by both the molecular chain arrangement and some radicals (such as oxygen) connected to the molecular chain^56^. XPS measurement has been conducted to characterize the electrostatic bonding for carbon atoms within the carbon framework. Figure S3 shows the wide energy survey spectra of all samples. In all four scans, the peaks observed at 284 eV and 530 eV are attributed to C1s and O1s, respectively. In the case of R1, the intensity of the oxygen peak is weaker than that of the others, indicating a lower level of carbon framework oxidation. Figure 8 shows the high resolution C1s spectra obtained from the four samples. All four C1s peaks can be deconvoluted into three components that correspond to carbon atoms in different functional groups, namely: the C in C=C bonds, the C in C-O bonds, and the C in C=O bonds located at 284.7, 285.4, and 288.4 eV, respectively.

These functional groups in C1s spectra (in Fig. 8) of the carbon framework were also confirmed by FTIR measurement, as shown in Fig. S4. Peaks located at 1760, 1605, 1385, and 1108 cm$^{-1}$ were found, which correspond to the C=O, C=C, C-OH, and C-O functional groups, respectively. The only notable difference in the XPS spectra of C1s was found for the peak located at 288.4 eV (C in C=O moieties). Its intensity shows an order of R1< R2< R3< R4, which indicates that the concentration of the C=O functional group is inversely proportional to the basic mechanical properties of the carbon framework of the rice culm. In addition, the relatively weak oxygen peak of R1 also confirmed this conclusion (see Fig. S3). The reverse effect of oxidation on the mechanical properties of the carbon framework is very common in physical chemistry because the oxidation groups can break the tough carbon chains, leading to the weakened mechanical strength of the related samples.

A graphical abstract of this study is presented in the Supporting Information, see Fig. S5.

## Conclusions

Four different rice cultivars were grown on the same farmland under same condition and for same duration. However, their lodging resistance was obviously different from each other. The results reveal that the mechanism behind this difference is a synergistic effect. First, with good agreement with previous studies, the mechanical properties of the culm are largely determined by the thickness of the culm wall. In particular, rather than the generally believed theory of the overall thickness of the fresh culm wall, the results in this study indicate that it is the depressed region of the dried basal culm wall that acts as the weak point, constraining the mechanical properties of the culm. Second, results indicate that the flexibility of the culm is more important than its rigidity for a better lodging resistance of the rice plants. The culm and its carbon framework always offer sufficient strength and rigidity for both support and stability of the rice stem. The constraint of the high lodging resistance of rice plants comes from the culm flexibility. Furthermore, based on the PALS measurement, the flexibility of the carbon framework is improved by (1) a more amorphous part, and (2) higher fractional free volume, because both of them govern higher mobility of the carbon molecular chain in the carbon framework. Third, in terms of chemical composition, relatively high nitrogen element content can benefit the growth and development of the rice culm. The relatively high degree of oxidation, in particular that of the C=O bond, is unfavorable for the fundamental mechanical properties of the carbon framework. Finally, as a conclusion, in the view of the guidance for management of the lodging stress, the possible methods recommended in this study to reduce the risk of lodging threat in rice can be obtained based on the following aspects: (1) the thickness of the depressed region of the culm wall should be increased, (2) the flexibility of culm and its carbon framework should be enhanced (by decreasing the crystallinity of the fresh culm and increasing the porous structure of its carbon framework), and (3) the N element content in the culm during the rice growth period should be adjusted. To the best of our knowledge, the conventional breeding is a possible approach to achieve the objectives mentioned as aspects (1) and (2).

## Supplementary Information

Supplementary Information.

## Data Availability

All data generated or analysed during this study are included in this published article [and its supplementary information files].
