# Supplementary material for: Lodging resistance of rice plants studied from the perspective of culm mechanical properties, carbon framework, free volume, and chemical composition
Source: Sci Rep. 2022 Nov 21;12:20026. doi: 10.1038/s41598-022-24714-4 (PMC9681888; doi:10.1038/s41598-022-24714-4)
Supplement: Supplementary file 1 — Supplementary Information. [file 41598_2022_24714_MOESM1_ESM.pdf]

Supporting Information

Lodging resistance of rice plants studied from the perspective of culm mechanical properties, carbon framework, free volume, and chemical composition

Qing Liu[a], Chongshan Yin[b*], Xi Li[b*], Chunqing He[c], Zhi Ding[b], Xuan Du[b]

[a] Institute of Subtropical Agriculture, Chinese Academy of Sciences, Changsha, China.

[b] Hunan Provincial Key Laboratory of Flexible Electronic Materials Genome Engineering, School of Physics and Electronic Science, Changsha University of Science and Technology, Changsha 410114, China.

[c] Key Laboratory of Nuclear Solid State Physics Hubei Province, School of Physics and Technology, Wuhan University, Wuhan 430072, China.

*E-mail: c.sh.yin@foxmail.com

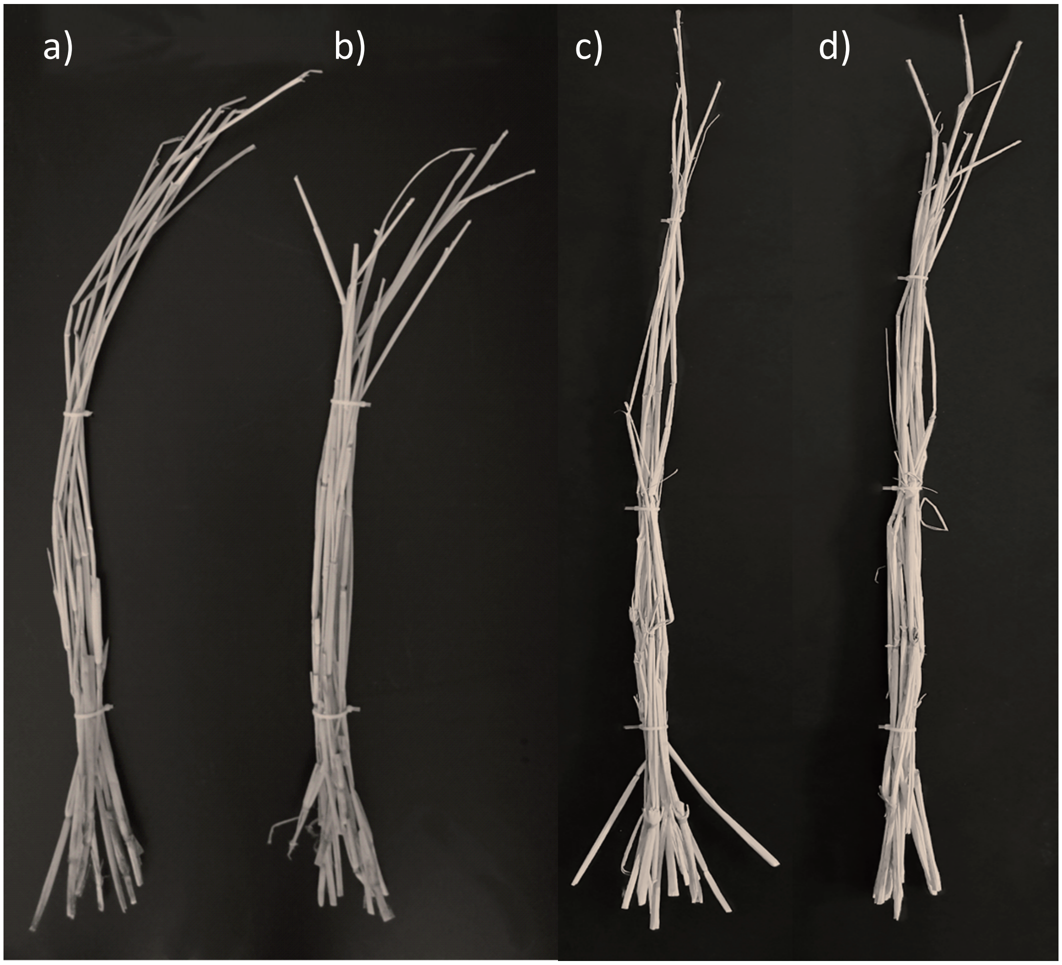

Fig. S 1 Pictures of the culm of a) R1, b) R2, c) R3, and d) R4.

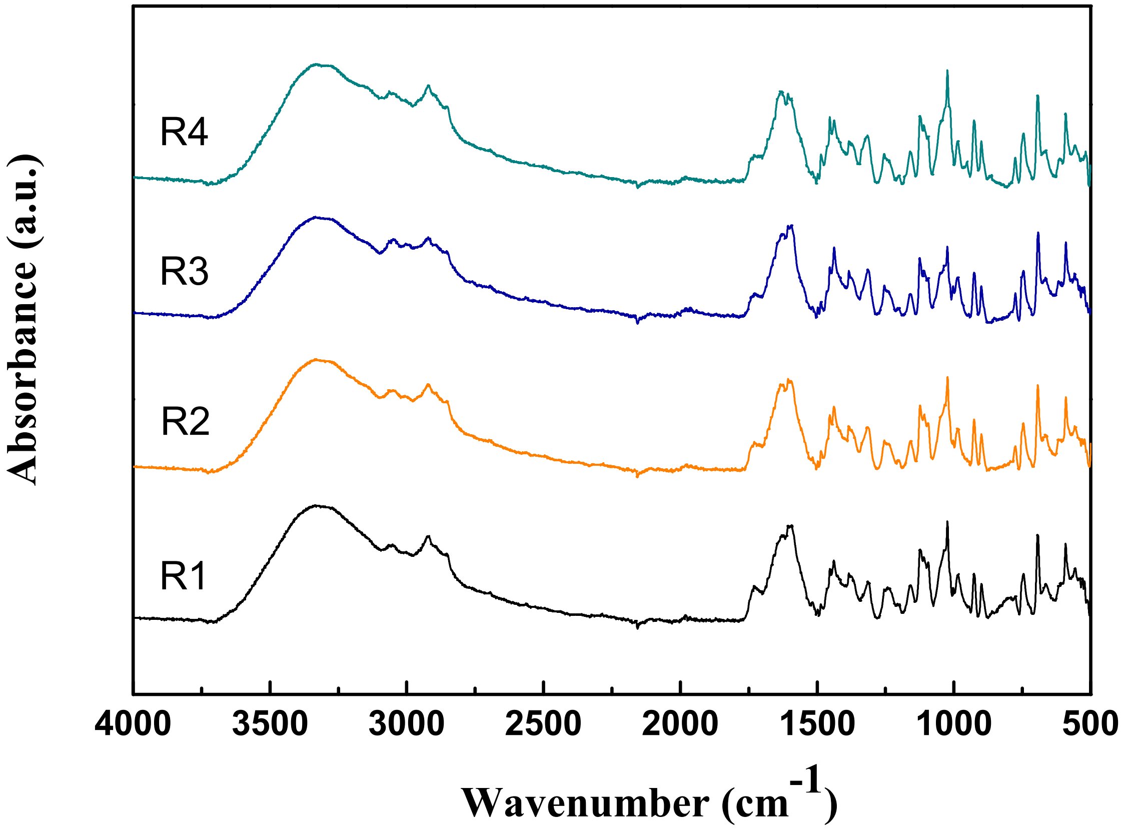

Fig. S 2 FTIR spectra of the powdered basal culm of the four varieties of rice plants.

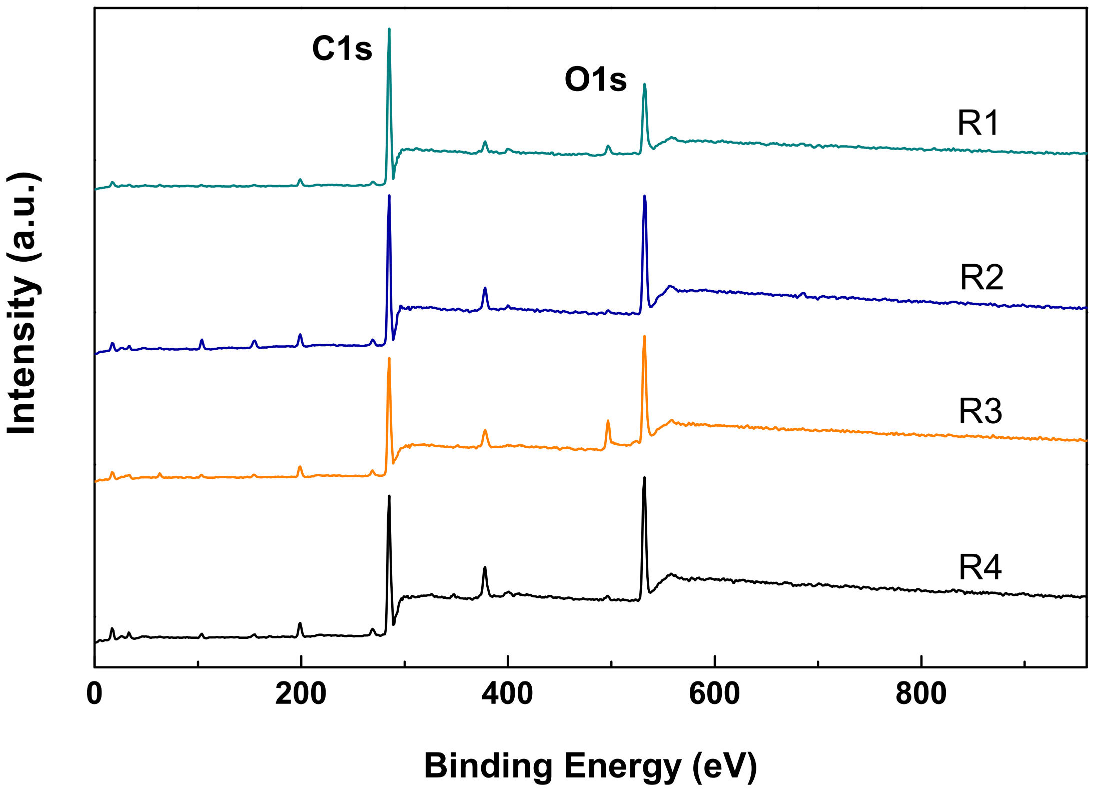

Fig. S 3 Wide XPS energy survey spectra of the powdered basal culm of the four varieties of rice plants.

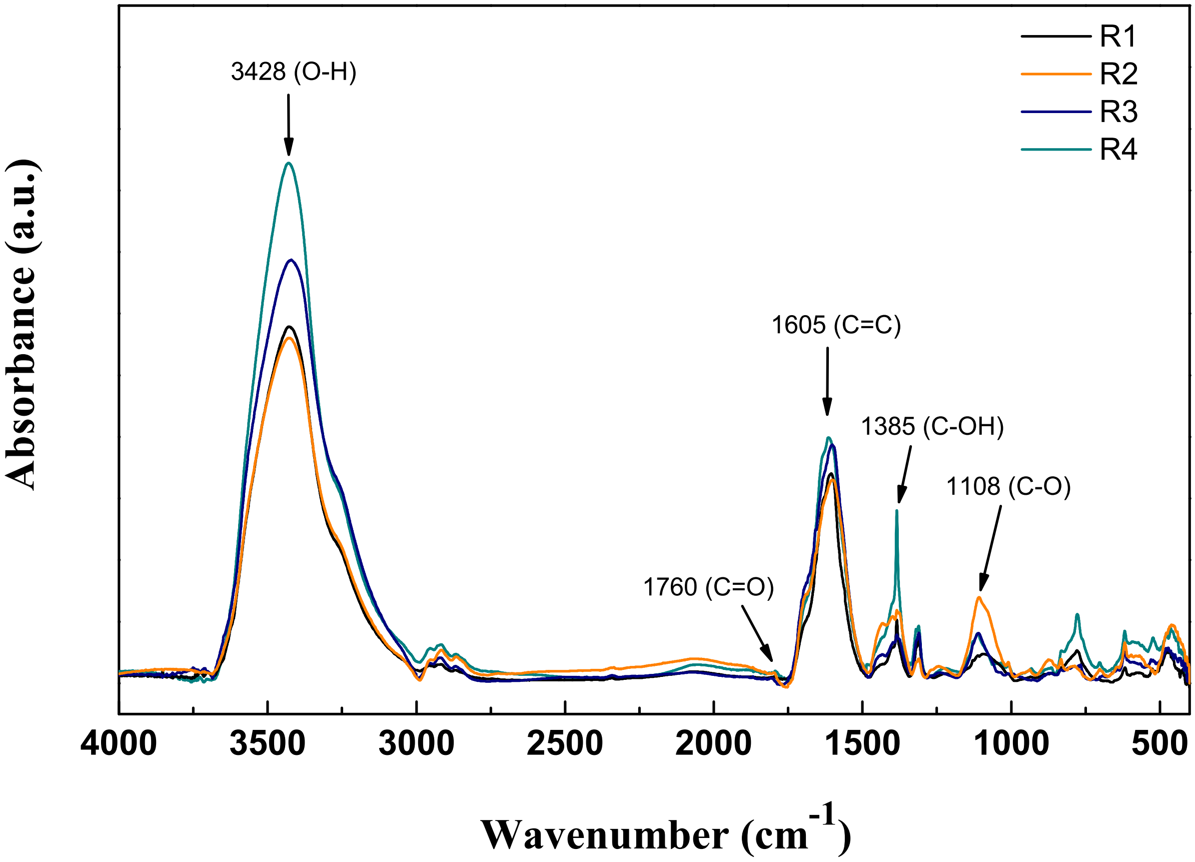

Fig. S 4 FTIR spectra of the carbon framework in basal culm of the four varieties of rice plants.

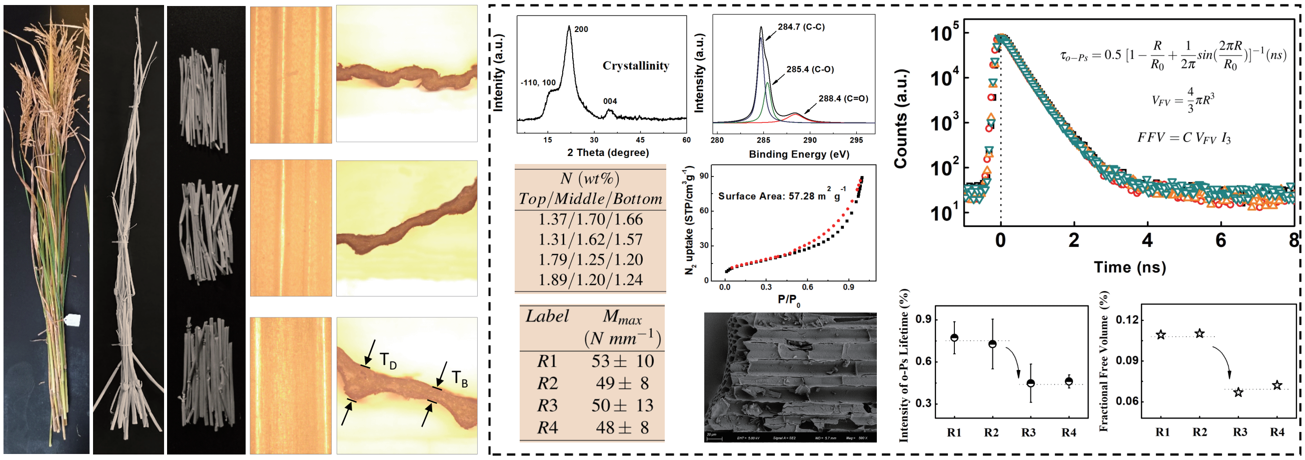

Fig. S 5 The graphical abstract of this work.

Mechanical property measurement (maximum bending moment)

The mechanical properties (maximum bending moment) of the basal culm was measured on a tensile machine (HDV, Yueqing Handpi Instruments, Co., Ltd., China). The fresh culms were made into the slender shape along the longitudinal direction. Three-point bending test is used to characterize the bending properties of rice culm. The samples were collected from the bottom of rice culm (between the node at bottom and the root). The bending test was carried out at a 50 mm support span. All samples were tested under the loading rate of 1 mm/min to obtain the maximum bending force.

Positron annihilation lifetime measurements

Before measurement, the carbonized rice culm was reduced into a fine powder and then mechanically pressed to tablets. The positron annihilation experiments were conducted by using a fast-fast coincidence PALS with a time resolution function of 0.230 ns for the full width at half maximum (FWHM), and 1 million counts were collected for each spectrum. A ^{22}Na source ($\sim$10 μCi) was firstly sandwiched by tablet samples (prepared with direct compression method) and then covered with an Al foil. PALS measurement was conducted under room temperature (25 °C) and room humidity ($\sim$80 %RH). The PATFIT program[1] as well as the LT program[2] were applied to analyse the resulting positron lifetime spectra, and the variances of the fits were in the range of 0.96$\sim$1.10. Details of PALS measurements set up can be found in our recent paper.[3]

Application of positron annihilation lifetime spectroscopy (PALS) to carbonized rice culm relies on the fact that a part of the injected positrons combine with electrons from surrounding molecules to form positronium (Ps) atoms in them. Ps is a hydrogen-like bound state of a positron and an electron with two spin states: spin antiparallel para-positronium (p-Ps) and spin parallel ortho-positronium (o-Ps). It's known that the intrinsic lifetime of o-Ps via 3γ annihilation under vacuum condition is 142 ns. However, being localized in free volume holes in materials, o-Ps undergoes 2γ pickup annihilation and its lifetime is shortened down to a few nanoseconds depending on the free volume hole size. Generally, positrons may annihilate via p-Ps, free positron and o-Ps. These three annihilation branches of positrons can be respectively characterized by their lifetimes τ_1 ($\sim$125 ps), τ_2 ($\sim$300 ps), τ_3 (1$\sim$10 ns) and the corresponding relative intensities. Being the longest lived component, the o-Ps lifetime (τ_{o-Ps}) is of particular importance for the studies of free volumes because it's related to the average free volume hole

size and it is determined by the overlapping of positronium wave function with that of the electrons on the wall of free volume holes.[4–7] For several decades, PALS has been widely used to measure atom-sized free volume holes in various solid materials. The average radii of hole free volumes in materials, which are determined by the semiempirical relationship between τ_{o-Ps} and mean free volume hole radius (R) in a spherical approximation given by the Tao-Eldrup model as,[4,5]

$$\tau_{o-Ps} = 0.5[1 - \frac{R}{R_0} + \frac{1}{2\pi} sin(\frac{2\pi R}{R_0})]^{-1} (ns) \tag{1}$$

where R_0=R+ΔR, and ΔR=0.166 nm is the thickness of the homogeneous electron layer overlapping with the o-Ps wave function. The mean hole free volume V_{FV} can be calculated from the following equation,

$$V_{FV} = \frac{4}{3}\pi R^3. \tag{2}$$

References

[1] Kirkegaard P., Pedersen N.J., & Eldrup, M.M., Patfit-88: A data-processing system for positron annihilation spectra on mainframe and personal computers. *J. Electr. Electron. Eng., Aust.*, **5**, 152-157 (1989).

[2] Kansy, J., Microcomputer program for analysis of positron annihilation lifetime spectra. *Nucl. Instr. Meth. A*, **374**, 235-244 (1996).

[3] Yin C., Li J., Zhou Y., Zhang H., Fang P., & He, C., Enhancement in proton conductivity and thermal stability in Nafion membranes induced by incorporation of sulfonated carbon nanotubes. *ACS Appl. Mater. Interfaces*, **10**, 14026-14035 (2018).

[4] Eldrup M., Lightbody D., & Sherwood J.N., The temperature dependence of positron lifetimes in solid pivalic acid. *Chem. Phys.*, **63**, 51-58 (1981).

[5] Tao S.J., Positronium annihilation in molecular substances. *J. Chem. Phys.* **56**, 5499-5510 (1972).

[6] Kobayashi Y., Zheng W., Meyer E.F., McGervey J.D., Jamieson A.M., & Simha, R., Free volume and physical aging of poly (vinyl acetate) studied by positron annihilation. *Macromolecules*, **22**, 2302-2306 (1989).

[7] Jean Y.C., Positron annihilation spectroscopy for chemical analysis: A novel probe for microstructural analysis of polymers. *Microchem. J.,* **42**, 72-102 (1990).
